# Shared susceptibility loci at 2q33 region for lung and esophageal cancers in high-incidence areas of esophageal cancer in northern China

**DOI:** 10.1371/journal.pone.0177504

**Published:** 2017-05-18

**Authors:** Xue Ke Zhao, Yi Min Mao, Hui Meng, Xin Song, Shou Jia Hu, Shuang Lv, Rang Cheng, Tang Juan Zhang, Xue Na Han, Jing Li Ren, Yi Jun Qi, Li Dong Wang

**Affiliations:** 1 Henan Key Laboratory for Esophageal Cancer Research, The First Affiliated Hospital of Zhengzhou University, Zhengzhou, Henan, China; 2 Henan Key Laboratory of Cancer Epigenetic, Cancer Institute, The First Affiliated Hospital, College of Clinical Medicine, Henan University of Science and Technology, Luoyang, Henan, China; Duke Cancer Institute, UNITED STATES

## Abstract

**Background:**

Cancers from lung and esophagus are the leading causes of cancer-related deaths in China and share many similarities in terms of histological type, risk factors and genetic variants. Recent genome-wide association studies (GWAS) in Chinese esophageal cancer patients have demonstrated six high-risk candidate single nucleotide polymorphisms (SNPs). Thus, the present study aimed to determine the risk of these SNPs predisposing to lung cancer in Chinese population.

**Methods:**

A total of 1170 lung cancer patients and 1530 normal subjects were enrolled in this study from high-incidence areas for esophageal cancer in Henan, northern China. Five milliliters of blood were collected from all subjects for genotyping. Genotyping of 20 high-risk SNP loci identified from genome-wide association studies (GWAS) on esophageal, lung and gastric cancers was performed using TaqMan allelic discrimination assays. Polymorphisms were examined for deviation from Hardy-Weinberg equilibrium (HWE) using Х^2^ test. Bonferroni correction was performed to correct the statistical significance of 20 SNPs with the risk of lung cancer. The Pearson’s Х^2^ test was used to compare the distributions of gender, TNM stage, histopathological type, smoking and family history by lung susceptibility genotypes. Kaplan-Meier and Cox regression analyses were carried out to evaluate the associations between genetic variants and overall survival.

**Results:**

Four of the 20 SNPs identified as high-risk SNPs in Chinese esophageal cancer showed increased risk for Chinese lung cancer, which included rs3769823 (OR = 1.26; 95% CI = 1.107–1.509; *P* = 0.02), rs10931936 (OR = 1.283; 95% CI = 1.100–1.495; *P* = 0.04), rs2244438 (OR = 1.294; 95% CI = 1.098–1.525; *P* = 0.04) and rs13016963 (OR = 1.268; 95% CI = 1.089–1.447; *P* = 0.04). All these SNPs were located at 2q33 region harboringgenes of *CASP8*, *ALS2CR12* and *TRAK2*. However, none of these susceptibility SNPs was observed to be significantly associated with gender, TNM stage, histopathological type, smoking, family history and overall survival.

**Conclusions:**

The present study identified four high-risk SNPs at 2q33 locus for Chinese lung cancer and demonstrated the shared susceptibility loci at 2q33 region for Chinese lung and esophageal cancers.

## Introduction

Cancers from lung and esophagus are the most common cancers and remain the leading causes of cancer-related deaths in China. For both cancers, squamous cell carcinoma is the predominant histopathological type and cigarette smoking and alcohol consumption are the main risk factors [1–5]. The concurrent occurrence of malignant lesions from lung and esophagus is not uncommon. Lung is the most common site for secondary primary malignancies in patients with esophageal cancer [4]. Accumulating evidence indicates that lung and esophageal cancers share many common genetic variants, such as deletion of *CASP* and *CYP2A6* gene [3, 6], *SOX2* expression [2], *IFGR*-IGFBP axis [7] and WDHD1 [8], especially in an Asian population.

Recently, a couple of genome-wide association studies (GWAS) demonstrated that a group of common genetic variants increase the risk to tumors from esophagus, lung and stomach [9–21]. Notably, the SNPs among these genetic variants have not been well characterized in lung cancer from Chinese Han population. Based on the published GWAS data from lung cancer, esophageal squamous cell carcinoma (ESCC) and gastric adenocarcinoma (GA), a group of 20 SNPs of interest were selected to determine their relevance on the susceptibility and prognosis in 1170 patients with lung cancer and 1530 healthy control subjects from northern China.

## Methods

### Ethics statement

The study was approved by the Ethics Committee of The First Affiliated Hospital of Zhengzhou University, China and conducted according to Declaration of Helsinki principles. Written informed consent was obtained from all participants.

### Patients and follow-up

For genotyping, 1170 lung cancer patients were enrolled in this study from January 1, 2011 to December 31, 2012, including 811 males with a mean age of 60±12 years (mean±SD) and 359 females with a mean age of 58±13 years. All patients were Chinese Han descent and were from multiple hospitals across Henan province, northern China, one of the high-incidence areas for both ESCC and GA. Detailed clinical information including histological type, lymph node metastasis, pathological stage, family history, cigarette smoking and treatment methods were retrieved from medical records. All patients were followed up until the end of 2014 with a median of 3.5 years. Survival status was available for all the patients genotyped in this study. In addition, 1530 healthy control subjects of Chinese Han descent were from the same regions of above lung cancer patients. 5ml peripheral venous blood was extracted from all the subjects enrolled in this study, blood DNA from all the subjects were retrospectively genotyped.

Family history was self-reported and defined as any first-degree relatives (parents or siblings) who had been diagnosed with lung cancer reported by the subjects. Positive smoking history was defined as more than 15 pack-years. Never smokers were individuals who reported smoking less than 100 cigarettes in their lifetime [22]. TNM stages were defined according to the seventh edition of the American Joint Committee on Cancer staging system, published in 2009 [23].

### DNA extraction and SNP genotyping

DNA was extracted from buffy coat of 1170 patients diagnosed with lung cancer and 1530 healthy controls using Puregene Kit (Qiagen, Valencia, CA, USA). All DNA samples were genotyped for the 20 susceptibility SNPs at 17 risk loci derived from lung cancer, ESCC or GA using TaqMan allelic discrimination assays (Applied Biosystems, Foster City, CA, USA). The reaction consisted of 2μl of 10 ng/μl DNA, 0.5μl of 2ⅹTaqman Universal PCR Master Mix, No AmpErase UNG (Bio-Rad, Hercules, CA, USA). The fluorescent intensities of each sample were measured before and after PCR using an Applied Biosystems 7500 Real-Time PCR System (Applied Biosystems). Data obtained was analyzed and genotypes assigned using 7300 System SDS Software, version 1.4 (Applied Biosystems). Because of less quantity and poor quality of DNA, 1005 effective genotypes with lung cancer and 1473 effective genotypes with healthy control were obtained in our study finally.

### Statistical analyses

All polymorphisms were examined for deviation from Hardy-Weinberg equilibrium (HWE) using Х^2^ test. Bonferroni correction was performed to correct the statistical significance of 20 SNPs with the risk of lung cancer. Clinical parameters were examined for associations with individual SNPs using Fisher’s Exact Test. Kaplan-Meier and Cox regression analyses were carried out to evaluate the associations between genetic variants and overall survival. The Pearson’s Х^2^ test was used to estimate the associations of gender, grade, tumor type, cigarette smoking and family history with lung susceptibility genotypes of selected SNPs.

## Results

### People characteristics

The information, including age and gender of the subjects involved in the present study, were summarized in Table 1. The mean ages for male and female lung cancer patients and healthy control were 60±12, 58±13 and 48±14, 49±12 years, respectively. The majority of the cases were males (811/1170, 69.32%). Cox regression analyses did not reveal association between risk loci and gender, age.

**Table 1:** Demographic characteristics of patients and healthy controls.

	Lung cancer	Healthy Controls
Age(Mean±SE)		
male	60±12	48±14
female	58±13	49±12
Sex(n)		
male	811	728
female	359	802
Total	1170	1530

### Lung cancer susceptibility genotypes

The distribution of genotypes in this patient cohort was shown in Table 2. Twenty SNPs in 17 genes (Tables 2 and 3) were analyzed for association with the risk of lung cancer. Finally, five SNPs (rs3769823, rs10931936, rs2244438, rs13016963, rs7578456) were significant association with the increased risk of lung cancer in the high-incidence area of ESCC in northern China with p value of 0.001, 0.002, 0.002, 0.002 and 0.007. However, after Bonferroni correction multiple comparisons, of the original five significant SNPs, one SNP (rs7578456) was identified without significant p value (0.14). The other four SNPs (rs3769823, rs2244438, rs10931936, and rs13016963) showed significant associations with the increased risk of lung cancer with p value of 0.02, 0.04, 0.04 and 0.04, respectively. The 95% CIs were shown in Table 2. High linkage disequilibrium SNPs, which map to a region including CASP8, ALS2CR12 and TRAK2, identified as high-risk SNPs in Chinese esophageal cancer, showed significant associations with the increased risk of lung cancer.

**Table 2:** Association of twenty genetic variants and risk of lung cancer.

Risk genotypes	F_ A	F_ U	*P*	Bonferroni correction *P*	OR	95%CI
rs3769823 rs10931936 rs2244438 rs13016963 rs7578456 rs36600 rs13042395 rs1926203 rs9841504 rs4254535 rs8034191 rs1530057 rs2352028 rs4975616 rs2808630 rs748404 rs753955 rs2909430 rs13361707 rs78378222	0.2923 0.2999 0.2470 0.3051 0.2727 0.1882 0.2883 0.1476 0.1413 0.2530 0.0494 0.0845 0.2011 0.1470 0.1332 0.0805 0.3548 0.0499 0.4926 0.1774	0.2422 0.2504 0.2023 0.2571 0.2319 0.2127 0.2634 0.1655 0.1246 0.2739 0.0417 0.0749 0.1885 0.1385 0.1538 0.0861 0.3464 0.0481 0.4921 0.0862	0.0010 0.0020 0.0020 0.0020 0.0070 0.0777 0.1086 0.1579 0.1590 0.1705 0.2873 0.3128 0.3602 0.4832 0.5105 0.5635 0.6139 0.8091 0.9797 0.5200	0.02 0.04 0.04 0.04 0.14 1.554 2.172 3.158 3.18 3.41 5.746 6.256 7.204 9.664 10.21 11.27 12.278 16.182 19.594 10.4	1.2920 1.2830 1.2940 1.2680 1.2420 0.8585 1.1330 0.8731 1.1560 0.8977 1.1950 1.1390 1.0840 1.0720 0.8442 0.9302 1.0370 1.0400 1.0020 0.8500	1.107–1.509 1.100–1.495 1.098–1.525 1.089–1.447 1.061–1.045 0.724–1.017 0.973–1.319 0.723–1.054 0.945–1.414 0.769–1.408 0.860–1.661 0.885–1.446 0.912–1.287 0.882–1.303 0.511–1.369 0.728–1.189 0.899–1.197 0.759–1.425 0.874–1.148 0.460–1.530

F_ A = Variant frequency of cancer loci; F_ U = Normal control variant frequency; OR = odds ratio.

**Table 3:** Distribution of SNPs genotyping in case (patients with esophageal, lung or gastric cancer) and controls (normal healthy subjects).

SNPs	Gene	Allele	MAF(n)		*P*	*OR*	95%*CI*		Reference number	Cancer type
			Cases	Controls						
rs36600	MTMR3	T/C	0.117(1978)	0.086(2540)	0.0000	1.38	1.21	1.58	[14]	LC
Rs3769823	CASP8	T/C	0.29(2961)	0.27(3400)	0.0001	1.29	1.18	1.38	[9]	EC
Rs2244438	TRAK2	G/A	0.25(2961)	0.23(3400)	0.000	1.35	1.26	1.49	[9]	EC
rs748404	TGM5	C/T	0.825(1447)	0.797(36256)	0.0005	1.08	1.33	1.20	[16]	LC
rs9841504	ZBTB20	G/C	0.09(4294)	0.14(5882)	0.000	0.58	0.47	0.72	[20]	GA
rs2909430	TP53	G/A	0.17(443)	0.10(547)	0.000	1.94	1.06	3.57	[18]	LC
rs78378222	TP53	A/C	0.21(1014)	0.19(1839)	0.0001	0.85	0.46	1.53	[19]	LC
rs10931936	CASP8	T/C	0.27(1974)	0.31(2617)	0.0000	1.29	1.19	1.40	[3]	EC
rs4254535	GKN2/GKN1	C/T	0.67(1452)	0.68(36203)	0.0000	0.97	0.89	1.05	[16]	LC
rs13042395	C20orf54	T/C	0.47(380)	0.48(380)	0.0000	0.98	0.72	1.33	[20]	EC
rs8034191	AGPHD1	C/T	0.21(467)	0.16(388)	0.0036	1.61	1.17	2.21	[15,17]	LC
rs7578456	TRAK2	A/G	0.24(1974)	0.28(2617)	0.0000	1.27	0.17	1.38	[11]	EC
rs2352028	GPC5	T/C	0.32(295)	0.35(598)	0.0000	0.89	0.66	1.20	[13]	LC
rs13016963	ALS2CR12	A/G	0.27(2961)	0.31(3400)	0.0000	1.29	1.19	1.40	[9]	EC
rs4975616	TERT/CLPTM1	G/A	0.42(239)	0.48(553)	0.001	0.56	0.40	0.80	[16]	LC
rs1530057	RBMS3	A/C	0.05(1429)	0.05(36224)	0.0000	0.01	0.83	1.23	[16]	LC
rs1926203	ACTA2	T/G	0.67(1466)	0.67(36273)	0.0000	0.99	0.90	1.08	[16]	LC
rs13361707	PRKAA1	T/C	0.57(4294)	0.48(5882)	0.0000	1.42	1.24	1.62	[20]	GA
rs2808630	CRPP1/CRP	C/T	0.40(96)	0.41(124)	0.0000	1.04	0.74	1.36	[16]	LC
rs753955	MIPEP/TNFRSF	C/T	0.33(2331)	0.28(3065)	0.000	1.24	1.14	1.35	[14]	LC

### Relationships between lung cancer susceptibility SNPs and overall survival

**Fig 1:**
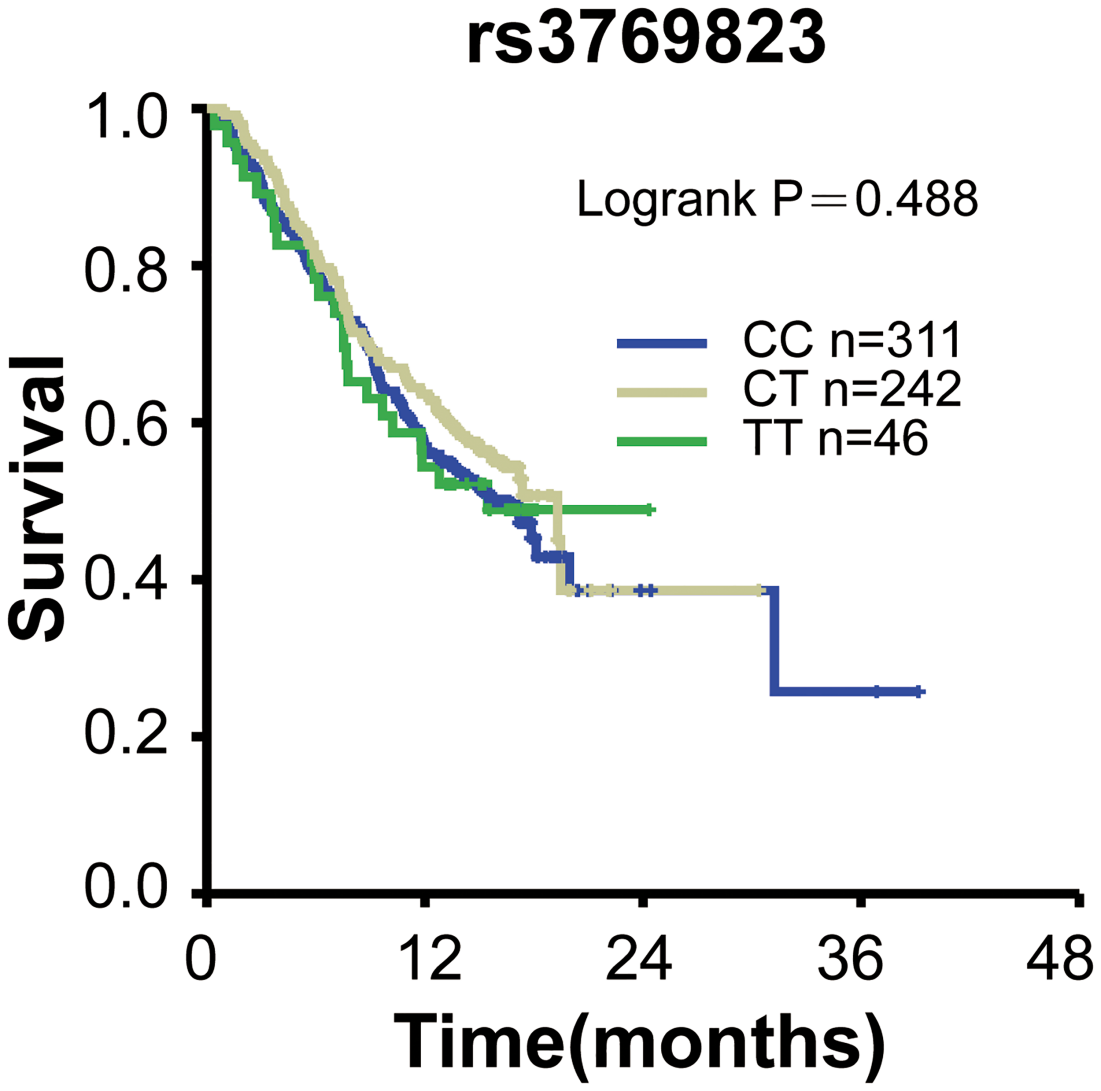
Kaplan-Meier survival curves with regards to rs3769823 genotypes.

**Fig 2:**
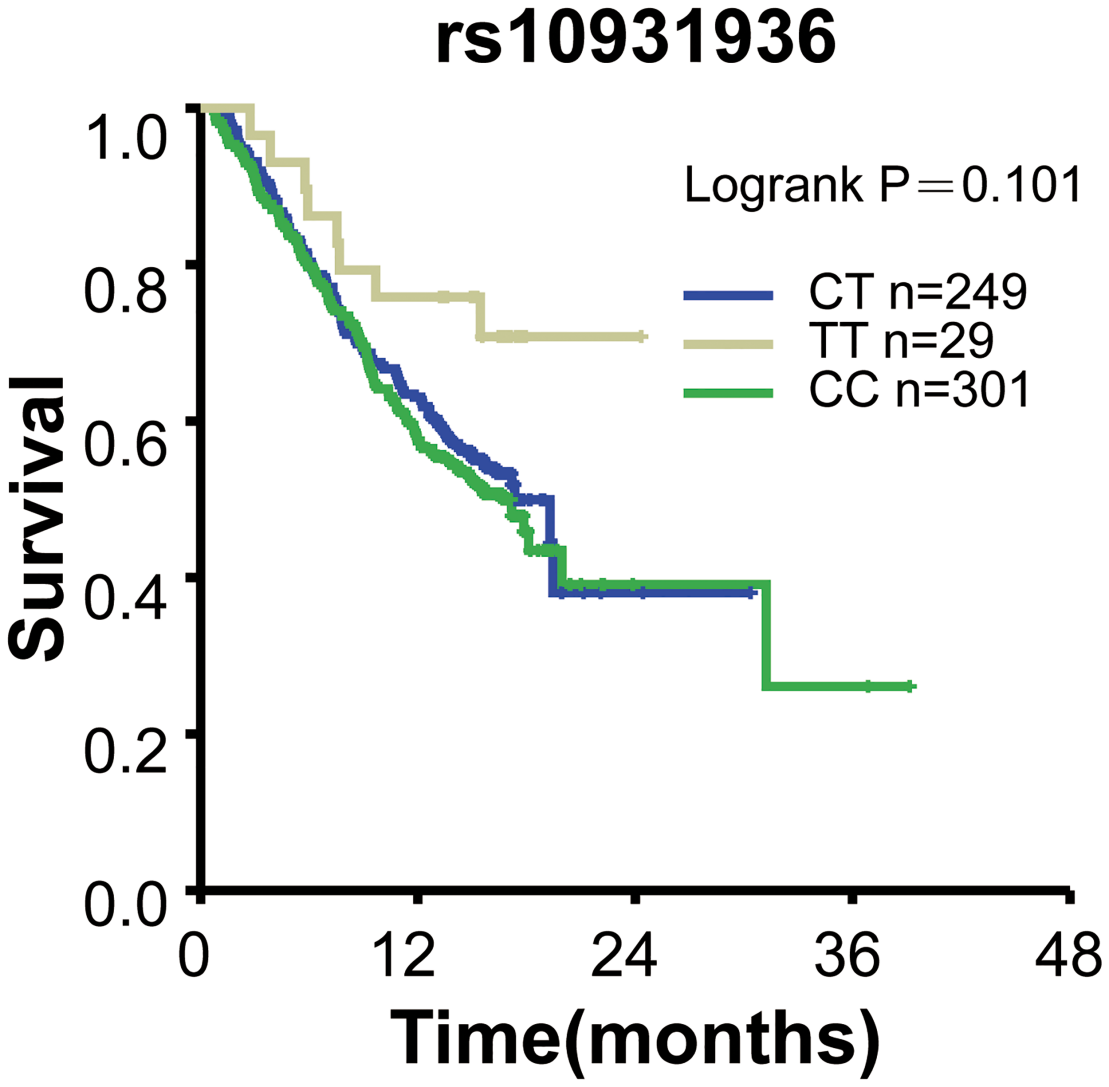
Kaplan-Meier survival curves with regards to rs10931936 genotypes. OS of the TT genotype group was increased compared with CT and AG genotype groups before expansion of abbreviation region.

**Fig 3:**
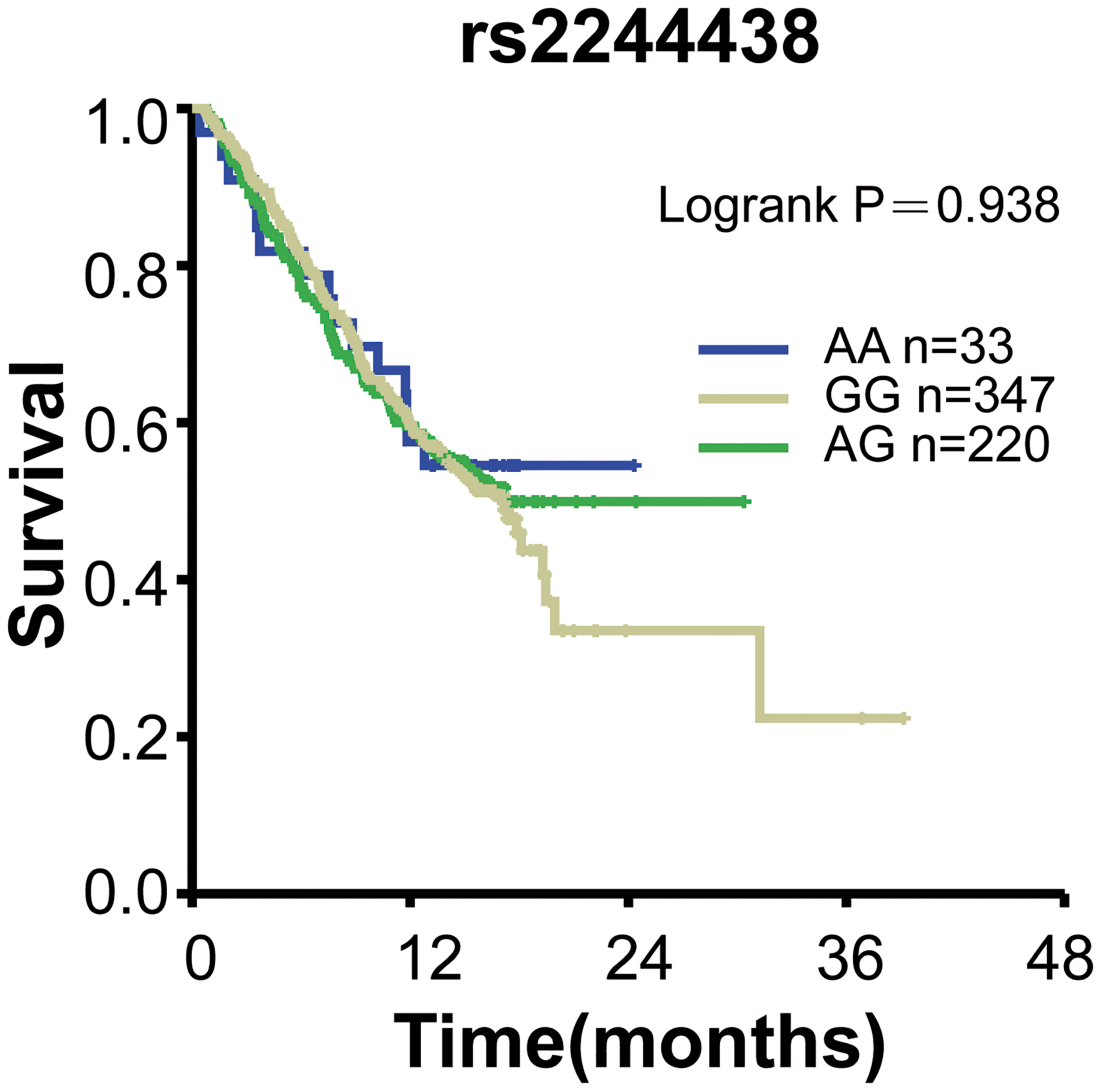
Kaplan-Meier survival curves with regards to rs2244438 genotypes.

**Fig 4:**
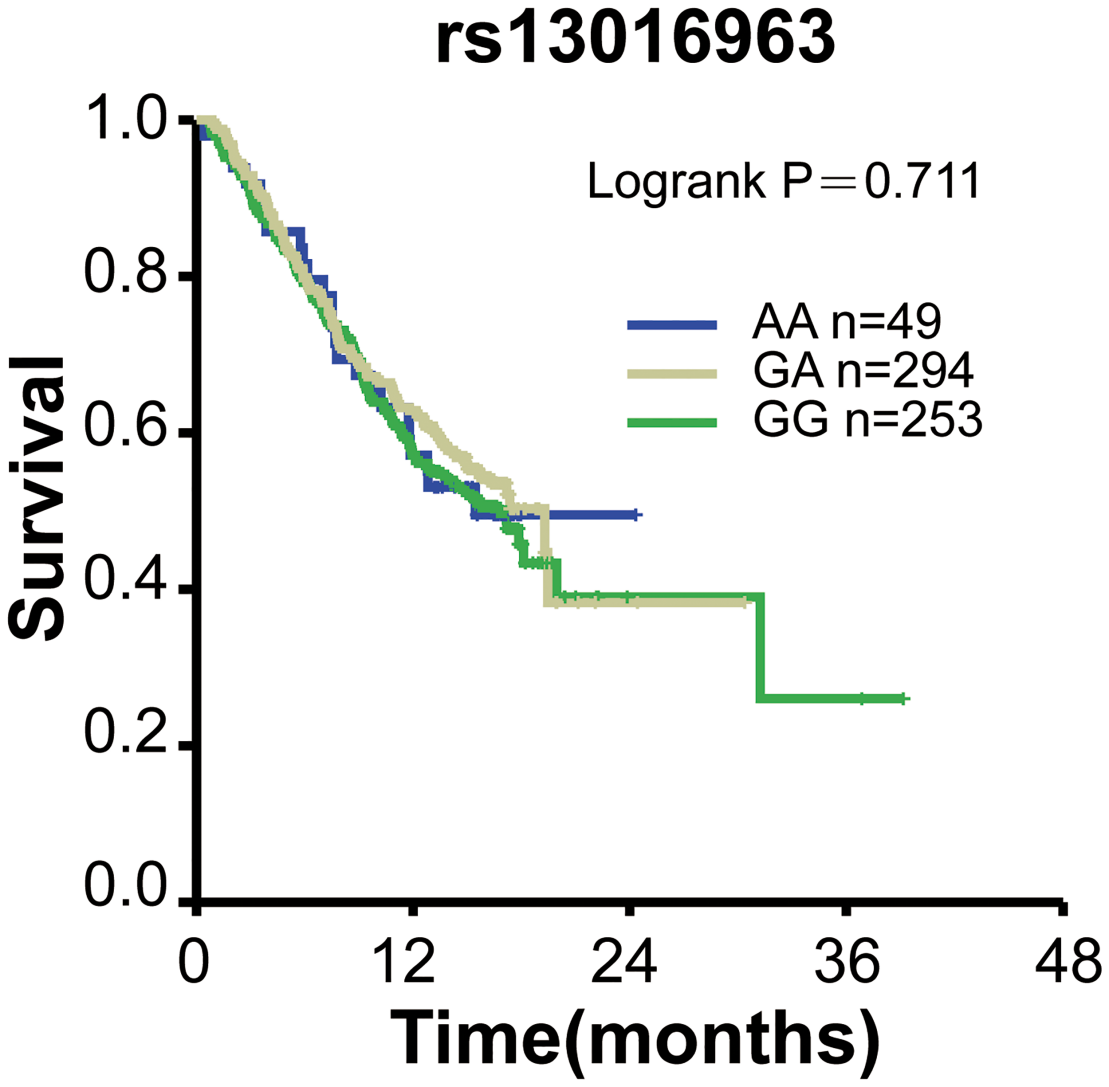
Kaplan-Meier survival curves with regards to rs13016963 genotypes.

To test whether these ESCC and lung cancer susceptibility SNPs variants correlate with the overall survival of lung cancer patients, we performed Kaplan-Meier survival analyses. Our results showed that no significant differences between overall survival (OS) and these SNPs were observed in patients (*P*>0.05; Table 4). 599 patients were genotyped for rs3769823, 579 for rs10931936, 600 for rs2244438, 596 for 13016963, respectively. Kaplan-Meier curve of OS for rs3769823 genotype was shown in Fig 1. Log-rank tests showed no significant difference between OS and rs10931936 genotype (log-rank *P* = 0.488). Fig 2 showed Kaplan-Meier curve of OS for rs10931936 genotype. OS of the TT genotype group increased in comparison with CT and AG genotype groups but without statistical significance. Kaplan-Meier curves of OS for rs2244438, rs13016963 genotypes were shown in Figs 3 and 4, respectively, and Log-rank analysis showed no significant differences between OS and these genotypes (log-rank *P* = 0.101, *P* = 0.938, *P* = 0.711, respectively).

**Table 4:** Association between overall survival and 4 risk genotypes of lung cancer.

SNP and genotype	Risk allele	Hazard ratio	95%CI	P	Case number		Total
					Yes/No	Total	
Rs3769823							
C/C	T	1.656	1.425–1.887	0.488	160/151	311	
T/T		1.286	1.064–1.509		23/23	46	599
T/C		1.517	1.340–1.694		111/131	242	
Rs10931936							
C/C	T	1.672	1.438–1.907	0.101	153/148	301	
T/T		1.624	1.383–1.866		8/21	29	579
T/C		1.499	0.324–1.673		117/132	249	
Rs2244438							
A/A	A	1.367	1.108–1.626	0.938	15/18	33	
A/G		1.576	1.439–1.713		104/116	220	600
G/G		1.609	1.383–1.835		176/171	347	
Rs1301663							
A/A	A	1.373	1.118–1.535	0.711	24/25	49	
A/G		1.501	1.326–1.676		118/13	253	596
G/G		1.667	1.432–1.902		150/144	294	

### Associations between the four risk genotypes of lung cancer and clinical characteristics

Table 5 summarizes the baseline characteristics of patients in this study. Among 1170 lung cancer patients, the main histopathological types were adenocarcinoma and squamous cell carcinoma. The majority of male patients of lung cancer were smokers. We found that rs10931936 CT genotype was borderline significantly associated with an increase risk in male subgroup (*P* = 0.086; Table 5). No statistically significant differences were found between four genotypes and gender, tumor types, grades, smoking and family history (*P*> 0.05, Table 5) when genotype and the allele distribution of polymorphisms were compared. The uninformative cases were due to lost of connection or low genome QC level.

**Table 5:** Association of four risk genotypes of lung cancer and clinical characteristics.

Characteristics	Rs22244438				Rs3769823				Rs10931936				Rs13016963			
	Total	GT	N (%)	*P* value	Total	GT	N (%)	*P* value	Total	GT	N (%)	*P* value	Total	GT	N (%)	*P* value
Gender																
Male	674	AA	41 (6)		674	CC	325 (48)		674	CT	296 (44)		674	AA	64 (9)	
		AG	263 (39)			TT	61 (9)			CC	316 (47)			GG	313 (47)	
		GG	370 (55)			TC	288 (43)			TT	62 (9)			GA	295 (43)	
Female	305	AA	18 (6)		305	CC	167 (55)		305	CT	113 (37)		305	AA	27 (9)	
		AG	104 (34)			TT	24 (8)			CC	166 (54)			GG	161 (53)	
		GG	183 (60)	0.311		TC	114 (37)	0.173		TT	26 (9)	0.086		GA	115 (38)	0.158
HPTP																
LAC	287	AA	19 (7)		287	CC	145 (50)		273	CT	115 (42)		286	AA	30 (10)	
		AG	110 (38)			TT	27 (9)			CC	143 (52)			GG	139 (49)	
		GG	158 (55)			TC	115 (41)			TT	15 (6)			GA	117 (41)	
LSC	201	AA	11 (6)		200	CC	100 (51)		188	CT	80 (42)		199	AA	19 (9)	
		AG	77 (38)			TT	18 (9)			CC	100 (53)			GG	98 (49)	
		GG	113 (56)			TC	82 (40)			TT	8 (5)			GA	82 (42)	
LSCC	153	AA	11 (7)		153	CC	82 (54)		153	CT	59 (41)		152	AA	19 (9)	
		AG	49 (32)			TT	13 (9)			CC	80 (55)			GG	79 (52)	
		GG	93 (61)			TC	58 (37)			TT	6 (4)			GA	59 (39)	
Other	47	AA	6 (13)		47	CC	22 (47)		47	CT	19 (46)		47	AA	10 (21)	
		AG	19 (40)			TT	10 (21)			CC	18 (44)			GG	19 (40)	
		GG	22 (47)	0.447		TC	15 (32)	0.237		TT	4 (10)	0.269		GA	18 (39)	0.592
TNM Stages																
I	19	AA	0 (0)		19	CC	11 (58)		19	CT	7 (37)		19	AA	1 (5)	
		AG	9 (47)			TT	1 (5)			CC	11 (58)			GG	10 (53	
		GG	10 (53)			TC	7 (37)			TT	1 (5)			GA	8 (42)	
II	24	AA	3 (13)		24	CC	8 (33)		22	CT	12 (55)		24	AA	4 (17)	
		AG	10 (41)			TT	4 (17)			CC	8 (36)			GG	8 (33)	
		GG	11 (46)			TC	12 (50)			TT	2 (9)			GA	12 (50)	
III	60	AA	4 (7)		60	CC	28 (47)		57	CT	27 (47)		59	AA	6 (10)	
		AG	24 (40)			TT	7 (12)			CC	27 (47)			GG	26 (44)	
		GG	32 (53)			TC	25 (41)			TT	3 (6)			GA	27 (46)	
IV	67	AA	5 (7)		67	CC	31 (46)		60	CT	26 (43)		60	AA	10 (15)	
		AG	23 (34)			TT	9 (13)			CC	31 (52)			GG	31 (46)	
		GG	39 (58)	0.565		TC	27 (41)	0.785		TT	3 (5)	0.678		GA	26 (39)	0.761
Smoking																
Yes	523	AA	31 (7)		522	CC	255 (49)		494	CT	227 (46)		484	AA	220 (46)	
		AG	203 (38)			TT	47 (9)			CC	247 (50)			GG	249 (51)	
		GG	289 (55)			TC	220 (42)			TT	20 (4)			GA	15 (3)	
No	389	AA	22 (6)		389	CC	205 (53)		374	CT	156 (42)		370	AA	149 (42)	
		AG	143 (37)			TT	28 (7)			CC	202 (54)			GG	207 (54)	
		GG	224 (57)	0.782		TC	156 (40)	0.444		TT	16 (4)	0.459		GA	14 (4)	0.467
Family history																
Yes	165	AA	7 (4)		165	CC	85 (52)		157	CT	69 (44)		165	AA	12 (7)	
		AG	55 (33)			TT	10 (6)			CC	83 (53)			GG	82 (50)	
		GG	103 (63)			TC	70 (42)			TT	5 (3)			GA	71 (43)	
No	745	AA	46 (6)		744	CC	369 (50)		708	CT	314 (44)		741	AA	71 (10)	
		AG	294 (40)			TT	68 (9)			CC	361 (51)			GG	354 (48)	
		GG	405 (54)	0.152		TC	307 (41)	0.414		TT	33 (5)	0.75		GA	316 (42)	0.64

HPTP = histopathological types; LAC = lung adenocarcinoma; LSC = lung squamous carcinoma; LSCC = lung small cell carcinoma; GT = genotype; Total = number of subjects examined

Other: other malignant tumor types of lung, hamartoma(3), carcinoma sareomatodes(1), undifferentiated carcinoma(3), adenosquamous carcinoma(14), mixed carcinoma(5), mucoepidermoid carcinoma(1), carcinoid tumor(2), tumor type undiagnosable(18)

## Discussion

In this study, we investigated the association between lung cancer and these functional polymorphisms at 2q33 predisposing to the risk of ESCC. The patients with lung cancer we studied came from high-incidence regions of ESCC, where exhibits a considerable high-incidence of lung cancer as well. Of all 20 SNPs, 6 SNPs were risk loci of ESCC, 12 SNPs were susceptibility loci of LC and only 2 SNPs were associated with GC. Interestingly, 4 of the 6 ESCC susceptibility SNPs showed significant association with LC in high-incidence areas of ESCC in northern China. All the ESCC patients associated with the six SNPs were Chinese people, and majority of them were from high-incidence area of ESCC in China. The fact may reveal lung cancer and ESCC may share many similar genetic variants. However, none of the 12 LC susceptibility SNPs showed significant association with LC in this study. Only 3 LC SNPs were from Chinese, other 9 of them were from foreigners. The result may indicate that there are significant regional and ethnic differences in susceptibility to lung cancer.

Our data revealed that four functional polymorphisms at 2q33 (rs13016963, rs3769823, rs10931936, rs2244438) out of 20 SNPs were associated with the risk of lung cancer (*P*< 0.01) and ESCC of Chinese Han as well. However, we did not find evidence of significant associations between overall survival of lung cancer and any of the four SNPs at 2q33.

Unlike highly penetrant germline mutations causing ‘hereditary’ cancers, such as *CDKN2A* mutations in melanoma and *BRCA1* mutations in breast cancer, lung cancer and ESCC belong to ‘sporadic’ (‘non-hereditary’) malignant phenotype but a number of recent studies of heritability assessment suggests that these phenotypes (include twelve cancer types) of sporadic tumors share common genetic variants [24]. In other words, the genetic variants in cancer susceptibility play important roles in several common cancers and more loci of remaining polygenic components should be discovered continuously. The effects of genes and common environmental factors on heritability should not simply be distinguished [24]. In fact, more novel cancer susceptibility loci identified by GWAS and contribution of all genetic variants to malignant disease should be simultaneously taken into consideration instead of just few known loci [9, 25, 26].

Numerous studies investigated whether variation in the *CASP8* [27, 28] gene region alters cancer risk, including cancers of lung [29], breast [30], pancreas [31], non-Hodgkin lymphoma [32], head and neck [3]. We identified two new loci (rs3769823, rs10931936) mapping to a genomic region harboring *CASP8* which associated with risk of both ESCC and lung cancer of Chinese Han. We are the first to report that SNPs mapping to *ALS2CR12* and *TRAK2* were significantly associated with ESCC and lung cancer of Chinese Han [33, 34]. Overall survival of allele rs10931936 TT genotype was increased compared with CT and CC genotype groups but without statistical significance (Fig 2, log-rank *P* = 0.101; Table 4). The cause may be due to the withdrawal of some cases from our follow-up and only informative 29 cases available for analysis. In addition, genotype CT in rs10931936 was borderline significantly associated with an increased risk in male subgroup (Table 5). Therefore, rs10931936 genotype group that map to *CASP8* need further investigation to elucidate [9]. Further research should focus on the association between functional polymorphisms at 2q33 (rs13016963, rs3769823, rs10931936, rs2244438) and risk of lung cancer from different ethnics, other tumor types and as well as the association with chemotherapy treatments in China.

So far, at least three studies on GWAS reported that rs8034191 was involved in the susceptibility to lung cancer [15–17]. However, our data from particular Chinese region found that rs8034191 was not associated with the risk of lung cancer. Furthermore, other investigations provided similar evidence that other SNPs may not modify the genetic risk of lung cancer, which include rs8034191, rs4975616, rs2352028, rs1926293 [35–38]. The discrepancy could be due to differences of ethnic, region, genotyping platform or study sizes.

The limitations of the present study are threefold. First, our analysis was only limited to a couple of regions in Northern China. Second, the sample size in our validation phase was smaller than the original studies. Third, the follow-up time was short for overall survival investigation.

## Conclusions

Our present results suggest that four genetic variants in 2q33 may contribute to the susceptibility to both lung and esophageal cancers in Han Chinese. However, we did not find an association between these four lung cancer risk variants with survival. In addition, our findings also showed that 16 common genetic variants that have been previously identified in lung cancer, ESCC or GA by GWAS were not associated with lung cancer susceptibility in the Han Chinese population.

## Supporting information

S1 File Genotypes of 4 SNPs for lung cancer patients and healthy control people. (ZIP)
